# Modeling and Analysis of an Energy-Efficient Mobility Management Scheme in IP-Based Wireless Networks^†^

**DOI:** 10.3390/s111211273

**Published:** 2011-11-28

**Authors:** Ho Young Hwang, Sun-Jong Kwon, Yun Won Chung, Dan Keun Sung, Suwon Park

**Affiliations:** 1 Department of Computer Engineering, Kwangwoon University, Seoul 139-701, Korea; E-Mail: hyhwang@kw.ac.kr; 2 Central R&D Laboratory, KT, Seoul 137-792, Korea; E-Mail: sunjong.kwon@kt.com; 3 School of Electronic Engineering, Soongsil University, Seoul 156-743, Korea; E-Mail: ywchung@ssu.ac.kr; 4 Department of Electrical Engineering, Korea Advanced Institute of Science and Technology, Daejeon 305-701, Korea; E-Mail: dksung@ee.kaist.ac.kr; 5 Department of Electronics and Communications Engineering, Kwangwoon University, Seoul 139-701, Korea

**Keywords:** energy-efficient mobility management, steady state analysis, Mobile IP

## Abstract

An energy-efficient mobility management scheme in IP-based wireless networks is proposed to reduce the battery power consumption of mobile hosts (MHs). The proposed scheme manages seven MH states, including transmitting, receiving, attention/cell-connected, attention/paging area(PA)-connected, idle, off/attached, and detached states, to efficiently manage battery power, radio resources, and network load. We derive the stationary probabilities and steady state probabilities of the seven MH states for the proposed scheme in IP-based wireless networks in compact form. The effects of various input parameters on MH steady state probabilities and power consumption are investigated in the proposed scheme compared to the conventional scheme. Network costs such as cell updates, PA updates, binding-lifetime-based registrations, and paging messages are analyzed in the proposed and conventional schemes. The optimal values of PA size and registration interval are derived to minimize the network cost of the proposed scheme. The combined network and power costs are investigated for the proposed and conventional schemes. The results provide guidelines to select the proper system parameters in IP-based wireless networks.

## 1. Introduction

Wireless networks and systems have evolved toward an IP-based network architecture. In such networks and systems, mobility needs to be handled at the IP layer based on the Internet Engineering Task Force (IETF) concept. Many IP-based mobility protocols, such as Mobile IPv4 (MIPv4) [1], Mobile IPv6 (MIPv6) [2], and IP micro-mobility protocols (e.g., Cellular IP [3] and HAWAII [4]) have been proposed and studied. MIPv4 [1] and MIPv6 [2] do not distinguish *idle* mobile hosts (MHs) from *active* MHs. These MIP protocols support registration but not paging. Hence, a care-of-address (CoA) needs to be updated whenever an MH moves to a different subnet which is served by a different foreign agent (FA) in the MIPv4 [1] or by a different access router (AR) in the MIPv6 [2] without regard to the MH states, *i.e.*, *active* and *idle*. This results in a significant waste of the MH battery power and an unnecessary signaling load because it is expected that wireless IP users are not actively communicating most of the time.

Various schemes on IP paging services for MHs have been studied. The P-MIP [5–6] is an extension to MIP which is proposed to reduce the signaling load in the core Internet. In this scheme, two MH states, *i.e.*, *active* and *idle*, were defined. In the *active* state of an MH, registration occurs whenever the MH changes its cell. On the other hand, in the *idle* state of an MH, a registration occurs only if the MH changes its paging area (PA). When there are any incoming data for the *idle* MH, paging is performed in order to find the exact location of the called MH.

Many enhancements for the MIP protocols, such as hierarchical Mobile IPv6 (HMIPv6) [7] and fast handover for Mobile IPv6 (FMIPv6) [8], have been investigated. Recently, the IETF Network-based Localized Mobility Management (NETLMM) working group proposed the NETLMM protocol [9–10]. The Proxy Mobile IPv6 (PMIPv6) [11] was also developed by the IETF NETLMM working group. In PMIPv6, the network supports IP mobility management on behalf of the MHs. Qualitative and quantitative comparisons between MIPv6 and PMIPv6 have been investigated by Kong *et al.* [12]. The PMIPv6-based global mobility management architecture and protocol procedure known as GPMIP was presented by Zhou *et al.* [13].

In our earlier study [14], we proposed a mobility management scheme that considered the *detached* and *off* states for IP-based mobile networks. We analyzed an optimal rate of binding-lifetime (BL)-based registrations which yields a minimum network cost when the registrations are utilized as a means of identifying the *off* state of MHs. To reduce MH power consumption, it is important to manage the *idle* MH state in the mobility management scheme for an efficient battery power management of MHs. In many IP-based mobility management schemes including that in our earlier study, MHs perform a PA-based registration in the *idle* or *dormant* state. However, to operate the MHs as a fully power-saving mode in the *idle* or *dormant* state, the state for the PA-based registration may need to be distinguished from the *idle* or *dormant* state.

In our earlier study [15], we proposed six MH states in which the communicating state was not divided into transmitting and receiving states. We derived the state transition probabilities and mean sojourn times in six MH states, and analyzed the effects of parameters based on simple exponential distribution assumed on session holding time and binding-lifetime. In our recent study [16], we derived the stationary probabilities and steady state probabilities of the proposed six MH states. The effects of session holding time on MH steady state probabilities and power consumption were investigated. We considered more practical distributions, *i.e.*, Erlang and Gamma distributions, on session holding time.

In this paper, we propose an energy-efficient mobility management scheme for reducing MH power consumption in IP-based wireless networks. The proposed scheme manages seven MH states, including transmitting, receiving, attention/cell-connected, attention/PA-connected, idle, off/attached, and detached states, to efficiently manage battery power, radio resources, and network load. To compare the power-saving effect of the proposed scheme with that of the conventional scheme, we derive the stationary probabilities and steady state probabilities of both the proposed scheme with the seven MH states and the conventional scheme for Mobile IP-based wireless networks in compact form. The effects of various input parameters on MH steady state probabilities and power consumption are investigated in both the proposed scheme and conventional scheme with consideration of exponential and fixed distributions on the interval of BL-based registration. Network costs such as cell updates, PA updates, binding-lifetime-based registrations, and paging messages are analyzed in the proposed and conventional schemes. The effects of various input parameters on the network costs for the proposed and conventional schemes are investigated. The optimal values of PA size and registration interval are derived to minimize the network cost of the proposed scheme. We also investigate the combined network and power cost for the proposed and conventional schemes using various weighting factors. These analytical results provide guidelines to select the proper system parameters. The results can be utilized to analyze the performance of mobility management schemes in IP-based wireless networks.

This paper is organized as follows: An IP-based wireless network architecture and an energy-efficient mobility management scheme are presented in Section 2. The MH state transitions are modeled, and the stationary probabilities and steady state probabilities of the seven MH states are analyzed. The MH energy consumptions as well as the network costs for both the proposed and conventional schemes are analyzed. The optimal values of PA size and registration interval are derived to minimize the network cost in Section 3. Numerical examples are used to investigate the MH steady state probabilities, the power saving effect, and the network costs compared with the conventional scheme for Mobile IP-based wireless networks in Section 4. Finally, conclusions are presented in Section 5.

## 2. IP-Based Wireless Network Architecture and MH State Transition Model

**Figure 1.**
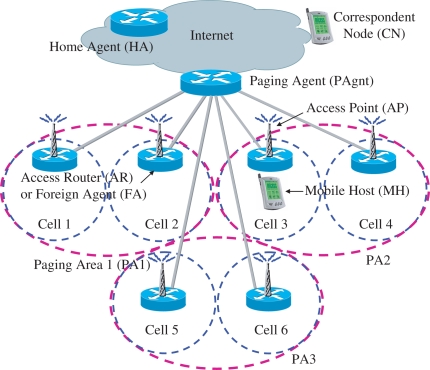
An IP-based wireless network architecture.

An IP-based wireless network architecture is shown in Figure 1 [15]. An access router (AR) provides MHs with IP connectivity. The AR acts as a default router to the currently served MHs. Since MIPv6 [2] provides many advantages over MIPv4 [1], MIPv6 [2] is considered a reference mobility protocol in this paper. However, we note that the proposed scheme can be applied to both the MIPv4 and MIPv6-based wireless networks. An energy-efficient mobility management scheme is proposed to manage the following seven MH states: transmitting, receiving, attention/cell-connected, attention/PA-connected, idle, off/attached, and detached states. Transmitting, receiving, and attention/cell-connected MHs behave in the same manner as MIP. The correspondent node (CN) and home agent (HA) do not need to be changed. The MH and paging agent (PAgnt) require only minor changes. The PAgnt conducts paging-related functions and manages one or more PAs. Two or more ARs can exist in a PA. To establish the PA identity, a unique PA identifier (PAI) can be used.

A transmitting, receiving, or attention/cell-connected MH registers its collocated care-of address (CCoA) at the corresponding HA as in MIP. Hence, the PAgnt does not need to be involved in the MIP registration procedure. When a transmitting, receiving, or attention/cell-connected MH moves to a different cell which is served by a different AR, the MH conducts cell-based registration in the same manner as the MIP registration. After the data session is completed, the MH enters the attention/cell-connected state and an attention timer is reset and restarted. The attention timer is used to decide the instant when the MH enters the idle state. If the attention timer expires, an attentive MH that is in the attention/cell-connected state or attention/PA-connected state enters the idle state by conducting PA-based registration. Through this PA-based registration, the MH can register a PAI of the current PA at the PAgnt. A paging agent care-of address (PAgnt-CoA) of the current PAgnt is registered at the corresponding HA of the MH. Whenever an idle MH moves to a different PA or PAgnt, the MH enters the attention/PA-connected state to conduct the PA-based registration and the attention timer is reset and restarted.

When data packets which are destined for an idle MH arrive at the HA, the packets are tunneled to the PAgnt. Hence, the HA is unaware of the idle state of the MH. The PAgnt buffers the data packets and sends paging request messages to ARs in the PA. The signaling messages can be sent to MHs via access points which are connected to the ARs. The corresponding idle MH enters the receiving state, and sends paging reply messages to the PAgnt. Concurrently, the MH registers its CCoA at the HA as the MIP registration. The PAgnt can forward the buffered data packets.

The MH power-off state can be detected by a BL-based registration and an unsuccessful paging. When the HA or the PAgnt sets a limitation on the maximum binding lifetime, the BL-based registration can be used to detect the power-off state of MHs. The network considers the MH state as detached when it detects a silence for more than an agreed time period or the MH does not respond to paging.

In the proposed scheme, an MH has the following seven states:

1. Transmitting: The MH registers its CCoA at the corresponding HA as MIP. In this state, the MH has outgoing sessions. The MH remains during the session holding time. The MH exits this state upon completing outgoing data sessions or a switch-off action.
2. Receiving: After the MH registers its CCoA at the corresponding HA as in MIP, the MH can receive the data packets. An exit from this state is caused by incoming session completion or switch-off action.
3. Attention/cell-connected: There is no incoming or outgoing session for the MH. The MH conducts a cell-based registration whenever it enters this state from the off/attached state or detached state or it changes its serving AR. Thus, the MH location is known in the network with cell accuracy. When an incoming or outgoing session arrives, the MH enters the transmitting or receiving state. When an attention timer expires, the MH enters the idle state by performing a PA-based registration.
4. Attention/PA-connected: The MH conducts the PA-based registration. Thus, the MH location is known in the network with PA accuracy. When an attention timer expires, the MH reenters the idle state. When an incoming or outgoing session arrives, the MH enters the communicating state. If the MH is switched off, it enters the off/attached state.
5. Idle: The MH is not currently involved in ongoing sessions and signaling messages. Thus, the idle MH can operate in a power-saving mode. When the idle MH moves to a different PA or PAgnt, the MH enters the attention/PA-connected state to perform the PA-based registration. When an MH is in the idle state, its current location information is maintained in terms of PA.
6. Off/attached: If the MH is powered off, the PAgnt is not immediately informed of the power-off state. The power-off state can be detected by a BL-based registration and an unsuccessful paging. When the binding-lifetime expires or paging is unsuccessful, the network detaches the MH. When the MH is switched on, it enters the attention/cell-connected state by performing the MIP registration.
7. Detached: If the network detects an MH switch-off action, it detaches the MH. The MH neither responds to paging nor sends location registration messages.

## 3. Analysis of an Energy-Efficient Mobility Management Scheme

### 3.1. Stationary Probabilities and Steady State Probabilities

**Figure 2.**
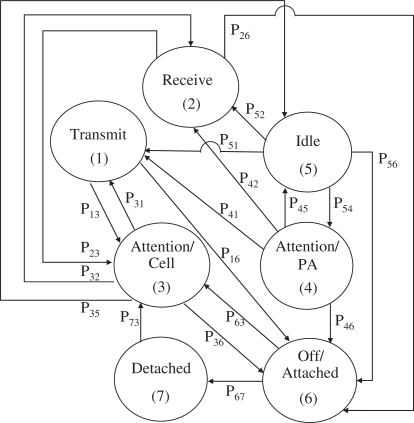
The mobile host (MH) state transitions.

We derive the stationary probabilities and steady state probabilities of seven MH states for the proposed energy-efficient mobility management scheme in IP-based wireless networks in compact form. MH state transitions are shown in Figure 2. We assume the following density functions of random variables:

- Incoming (receiving) and outgoing (transmitting) sessions occur at an MH according to a Poisson process with parameters λ*_i_* and λ*_o_*, respectively.
- The cell and PA residence durations are exponentially distributed with parameters 1/λ*_c_* and 1/λ*_PA_*, respectively.
- Switch-off actions take place according to a Poisson process with a parameter of λ*_off_*.
- The duration that an MH remains switched-off follows an exponential distribution with a parameter of 1/μ*_off_* .

Since the residence time of the MH in each state is not exponentially distributed, we analyze the MH state transitions using a semi-Markov process approach [15–17]. The stationary probabilities of the imbedded Markov chain are obtained by solving the balancing equations:

(1) $$\pi_{j}=\sum_{i=1}^{7}\pi_{i}P_{\mathrm{ij}},\;\;\;\;\;\;j=1,2,\ldots ,7$$

(2) $$1=\sum_{i=1}^{7}\pi_{i}$$

where *π_i_* denotes the stationary probability of state *i* while *P_ij_* denotes the state transition probability from state *i* to state *j*. The state transition probability matrix *P* = [*P_ij_*] for the MH state transitions is expressed as:

(3) $$P=\left(\begin{array}{ccccccc} 0 & 0 & P_{13} & 0 & 0 & P_{16} & 0\\ 0 & 0 & P_{23} & 0 & 0 & P_{26} & 0\\ P_{31} & P_{32} & 0 & 0 & P_{35} & P_{36} & 0\\ P_{41} & P_{42} & 0 & 0 & P_{45} & P_{46} & 0\\ P_{51} & P_{52} & 0 & P_{54} & 0 & P_{56} & 0\\ 0 & 0 & P_{63} & 0 & 0 & 0 & P_{67}\\ 0 & 0 & P_{73} & 0 & 0 & 0 & 0 \end{array}\right)$$

From Equations (1–3) the stationary probabilities of the seven MH states are solved as:

(4) $$\pi_{1}=\frac{1}{D}\left[\left\{(P_{31}\left(1-P_{45}P_{54}\right)+P_{35}\left(P_{41}P_{54}+P_{51}\right)\right\}\left(P_{63}+P_{73}P_{67}\right)\right]$$

(5) $$\pi_{2}=\frac{1}{D}\left[\left\{(P_{32}\left(1-P_{45}P_{54}\right)+P_{35}\left(P_{42}P_{54}+P_{52}\right)\right\}\left(P_{63}+P_{73}P_{67}\right)]\right]$$

(6) $$\pi_{3}=\frac{1}{D}\left[\left(1-P_{45}P_{54}\right)\left(P_{63}+P_{73}P_{67}\right)\right]$$

(7) $$\pi_{4}=\frac{1}{D}\left[P_{35}P_{54}\left(P_{63}+P_{73}P_{67}\right)\right]$$

(8) $$\pi_{5}=\frac{1}{D}\left[P_{35}\left(P_{63}+P_{73}P_{67}\right)\right]$$

(9) $$\pi_{6}=\frac{1}{D}\left[\left(1-P_{13}P_{31}-P_{23}P_{32}\right)\left(1-P_{45}P_{54}\right)-P_{13}P_{35}\left(P_{51}+P_{41}P_{54}\right)-P_{23}P_{35}\left(P_{52}+P_{42}P_{54}\right)\right]$$

(10) $$\pi_{7}=\frac{1}{D}\left[P_{67}\left\{\left(1-P_{13}P_{31}-P_{23}P_{32}\right)\left(1-P_{45}P_{54}\right)-P_{13}P_{35}\left(P_{51}+P_{41}P_{54}\right)-P_{23}P_{35}\left(P_{52}+P_{42}P_{54}\right)\right\}\right]$$

where *D* consists of the state transition probabilities *P_ij_*.

The steady state probabilities of the semi-Markov process are obtained by:

(11) $$P_{i}=\frac{\pi_{i}{\bar{T}}_{i}}{\sum {}_{j=1}^{7}\pi_{j}{\bar{T}}_{j}},\;\;\;\;i=1,2,\ldots ,7$$

where the values of the mean sojourn times of the MH in each state *i*, *T̄_i_* are expressed as [15]:

(12) $$\begin{array}{ll} {\bar{T}}_{1}=\frac{1-F_{s}^{*}\left(\lambda_{\mathrm{off}}\right)}{\lambda_{\mathrm{off}}}, & {\bar{T}}_{2}=\frac{1-F_{s}^{*}\left(\lambda_{\mathrm{off}}\right)}{\lambda_{\mathrm{off}}} \end{array}$$

(13) $$\begin{array}{ll} {\bar{T}}_{3}=\frac{1-e^{-\left(\lambda_{i}+\lambda_{o}+\lambda_{\mathrm{off}}+\lambda_{c}\right)T_{A}}}{\lambda_{i}+\lambda_{o}+\lambda_{\mathrm{off}}+\lambda_{c}e^{-\left(\lambda_{i}+\lambda_{o}+\lambda_{\mathrm{off}}+\lambda_{c}\right)T_{A}}}, & {\bar{T}}_{4}=\frac{1-e^{-\left(\lambda_{i}+\lambda_{o}+\lambda_{\mathrm{off}}\right)T_{A}}}{\lambda_{i}+\lambda_{o}+\lambda_{\mathrm{off}}} \end{array}$$

(14) $$\begin{array}{ll} {\bar{T}}_{5}=\frac{1-F_{r}^{*}\left(\lambda_{i}+\lambda_{o}+\lambda_{\mathrm{off}}+\lambda_{\mathrm{PA}}\right)}{\lambda_{i}+\lambda_{o}+\lambda_{\mathrm{off}}+\lambda_{\mathrm{PA}}}, & {\bar{T}}_{6}=\frac{1}{\lambda_{i}+\mu_{\mathrm{off}}}\left\{1-\frac{\lambda r}{\lambda_{i}+\mu_{\mathrm{off}}}\left[1-F_{r}^{*}\left(\lambda_{i}+\mu_{\mathrm{off}}\right)\right]\right\} \end{array}$$

(15) $${\bar{T}}_{7}=\frac{1}{\mu_{\mathrm{off}}}$$

The steady state probabilities of the seven MH states for the proposed scheme are derived in compact form as [16]:

(16) $$P_{1}=\frac{\mu_{\mathrm{off}}}{\lambda_{\mathrm{off}}+\mu_{\mathrm{off}}}\frac{\lambda_{o}\left(1-F_{s}^{*}\left(\lambda_{\mathrm{off}}\right)\right)}{\lambda_{s}+\lambda_{\mathrm{off}}}{\left[1-\frac{\lambda_{s}}{\lambda_{s}+\lambda_{\mathrm{off}}}F_{s}^{*}\left(\lambda_{\mathrm{off}}\right)\right]}^{-1}$$

(17) $$P_{2}=\frac{\mu_{\mathrm{off}}}{\lambda_{\mathrm{off}}+\mu_{\mathrm{off}}}\frac{\lambda_{i}\left(1-F_{s}^{*}\left(\lambda_{\mathrm{off}}\right)\right)}{\lambda_{s}+\lambda_{\mathrm{off}}}{\left[1-\frac{\lambda_{s}}{\lambda_{s}+\lambda_{\mathrm{off}}}F_{s}^{*}\left(\lambda_{\mathrm{off}}\right)\right]}^{-1}$$

(18) $$P_{3}=\frac{\lambda_{\mathrm{off}}\mu_{\mathrm{off}}}{\lambda_{\mathrm{off}}+\mu_{\mathrm{off}}}\frac{1-e^{-\lambda_{2}}}{\lambda_{1}+\lambda_{c}e^{-\lambda_{2}}}{\left[1-\frac{\lambda_{s}}{\lambda_{s}+\lambda_{\mathrm{off}}}F_{s}^{*}\left(\lambda_{\mathrm{off}}\right)\right]}^{-1}$$

(19) $$\begin{array}{l} P_{4}=\frac{\lambda_{\mathrm{off}}\mu_{\mathrm{off}}}{\lambda_{\mathrm{off}}+\mu_{\mathrm{off}}}\frac{\lambda_{1}F_{r}^{*}\left(\lambda_{1}+\lambda_{\mathrm{PA}}\right)+\lambda_{\mathrm{PA}}}{\lambda_{1}\left[1-e^{-\lambda 3}F_{r}^{*}\left(\lambda_{1}+\lambda_{\mathrm{PA}}\right)\right]+\lambda_{\mathrm{PA}}\left(1-e^{-\lambda_{3}}\right)}\\ \;\;\;\;\;\;\;\;\;\;\cdot \frac{\left(\lambda_{1}+\lambda_{c}\right)\left(1-e^{-\lambda_{3}}\right)e^{-\lambda_{2}}}{\lambda_{1}\left(\lambda_{1}+\lambda_{c}e^{-\lambda_{2}}\right)}{\left[1-\frac{\lambda_{s}}{\lambda_{s}+\lambda_{\mathrm{off}}}F_{s}^{*}\left(\lambda_{\mathrm{off}}\right)\right]}^{-1} \end{array}$$

(20) $$\begin{array}{l} P_{5}=\frac{\lambda_{\mathrm{off}}\mu_{\mathrm{off}}}{\lambda_{\mathrm{off}}+\mu_{\mathrm{off}}}\frac{1-F_{r}^{*}\left(\lambda_{1}+\lambda_{\mathrm{PA}}\right)}{\lambda_{1}\left[1-e^{-\lambda 3}F_{r}^{*}\left(\lambda_{1}+\lambda_{\mathrm{PA}}\right)\right]+\lambda_{\mathrm{PA}}\left(1-e^{-\lambda_{3}}\right)}\\ \;\;\;\;\;\;\;\;\;\;\cdot \frac{\left(\lambda_{1}+\lambda_{c}\right)e^{-\lambda_{2}}}{\lambda_{1}+\lambda_{c}e^{-\lambda_{2}}}{\left[1-\frac{\lambda_{s}}{\lambda_{s}+\lambda_{\mathrm{off}}}F_{s}^{*}\left(\lambda_{\mathrm{off}}\right)\right]}^{-1} \end{array}$$

(21) $$P_{6}=\frac{1}{\lambda_{i}+\mu_{\mathrm{off}}}\frac{\lambda_{\mathrm{off}}\mu_{\mathrm{off}}}{\lambda_{\mathrm{off}}+\mu_{\mathrm{off}}}\left[1-\frac{\lambda_{r}}{\lambda_{i}+\mu_{\mathrm{off}}}\left(1-F_{r}^{*}\left(\lambda_{i}+\mu_{\mathrm{off}}\right)\right)\right]$$

(22) $$P_{7}=\frac{1}{\lambda_{i}+\mu_{\mathrm{off}}}\frac{\lambda_{\mathrm{off}}}{\lambda_{\mathrm{off}}+\mu_{\mathrm{off}}}\left[\lambda_{i}+\frac{\lambda_{r}\mu_{\mathrm{off}}}{\lambda_{i}+\mu_{\mathrm{off}}}\left(1-F_{r}^{*}\left(\lambda_{i}+\mu_{\mathrm{off}}\right)\right)\right]$$

where λ_1_ = λ*_i_* + λ*_o_* + λ*_off_*, λ_2_ = (λ_1_ + λ*_c_*)*T_A_*, λ_3_ = λ_1_*T_A_*, λ*_s_* = λ*_i_* + λ*_o_*. λ*_i_* denotes the mean arrival rate of incoming sessions, λ*_o_* denotes the mean arrival rate of outgoing sessions, 1/λ_*c*_ denotes the mean cell residence duration, 1/λ*_PA_* denotes the mean PA residence duration, λ*_off_* denotes the mean switch-off rate, 1/μ*_off_* denotes the mean switch-off duration, and *T_A_* denotes the attention timer value. The session holding time is assumed to follow a general distribution with a density function *f_s_*(*t*) with a mean 1/μ*_s_* while $F_{s}^{*}(\theta )$ is the Laplace transform of *f_s_*(*t*). Since the Gamma distribution has the same trend as a Pareto distribution in terms of variance impact, it is useful for data packet transmission times [18–21]. The Erlang and Gamma distributions can be used for the session holding time. The interval of BL-based registration is assumed to follow a general distribution with a density function *f_r_*(*t*) with a mean of 1/λ*_r_* while $F_{r}^{*}(\theta )$ is the Laplace transform of *f_r_*(*t*).

### 3.2. The Power-Saving Effect

The power-saving effect of the proposed scheme is analyzed compared to the conventional scheme for Mobile IP-based wireless networks. To analyze the power-saving effect, let *Pc_i_* denote power consumption in state *i*. The energy consumption for the proposed scheme is obtained by:

(23) $$E_{\mathrm{prop}}=\sum_{i=1}^{5}Pc_{i}P_{i}t$$

When an *idle* MH moves to a different PA or PAgnt, the MH enters the *attention/PA-connected* state to perform the PA-based registration. The *idle* MH enters the *communicating* state when either an incoming or outgoing session arrives. Thus, the *idle* MH can operate in a power-saving mode since the *idle* MH may not have communicating sessions or signaling messages. The power consumption in each state of the MH has the following condition:

(24) $$Pc_{1}\geq Pc_{2}\geq Pc_{3}\geq Pc_{4}\gg Pc_{5}$$

Since the conventional MIP protocol [1–2] supports registration but not paging, it does not distinguish active MHs from idle ones. Hence, the value of the attention timer *T_A_* approaches *∞*. Therefore, the steady state probabilities for the conventional MIP scheme are expressed as:

$$\begin{array}{l} \underset{T_{A}\to \infty }{\text{lim}}P_{1}=P_{1},\;\;\;\;\;\underset{T_{A}\to \infty }{\text{lim}}P_{2}=P_{2}\\ \underset{T_{A}\to \infty }{\text{lim}}P_{3}={\left[1-\frac{\lambda_{s}}{\lambda_{s}+\lambda_{\mathrm{off}}}F_{s}^{*}\left(\lambda_{\mathrm{off}}\right)\right]}^{-1}\frac{\lambda_{\mathrm{off}}\mu_{\mathrm{off}}}{\lambda_{\mathrm{off}}+\mu_{\mathrm{off}}}\frac{1}{\lambda_{1}}\\ \underset{T_{A}\to \infty }{\text{lim}}P_{4}=0,\;\;\;\;\;\underset{T_{A}\to \infty }{\text{lim}}P_{5}=0\\ \underset{T_{A}\to \infty }{\text{lim}}P_{6}=P_{6}\frac{\lambda_{\mathrm{off}}\mu_{\mathrm{off}}}{\lambda_{\mathrm{off}}+\mu_{\mathrm{off}}}\frac{1}{\lambda_{i}+\mu_{\mathrm{off}}}\left[1-\frac{\lambda_{r}}{\lambda_{i}+\mu_{\mathrm{off}}}\left(1-F_{r}^{*}\left(\lambda_{i}+\mu_{\mathrm{off}}\right)\right)\right]\\ \underset{T_{A}\to \infty }{\text{lim}}P_{7}=P_{7}\frac{\lambda_{\mathrm{off}}}{\lambda_{\mathrm{off}}+\mu_{\mathrm{off}}}\frac{1}{\lambda_{i}+\mu_{\mathrm{off}}}\left[\lambda_{i}+\frac{\lambda_{r}\mu_{\mathrm{off}}}{\lambda_{i}+\mu_{\mathrm{off}}}\left(1-F_{r}^{*}\left(\lambda_{i}+\mu_{\mathrm{off}}\right)\right)\right] \end{array}$$

Then, the energy consumption for the conventional MIP scheme is obtained by:

(25) $$\begin{array}{ll} E_{\mathrm{conv}} & =\;\;\;\underset{T_{A}\to \infty }{\text{lim}}\sum_{i=1}^{5}Pc_{i}P_{i}t=\left(Pc_{1}P_{1}+Pc_{2}P_{2}+Pc_{3}\underset{T_{A}\to \infty }{\text{lim}}P_{3}\right)t\\ & =\;\;\;\left\{Pc_{1}P_{1}+Pc_{2}P_{2}+Pc_{3}\left(P_{3}+P_{4}+P_{5}\right)\right\}t \end{array}$$

From Equations (23–25), the relationship of the energy consumption between the proposed and conventional schemes is expressed as *E_prop_* *≤ E_conv_*. Therefore, the energy consumption for the proposed scheme is always lower than that for the conventional scheme. Additionally, the proposed scheme with a proper value of attention timer *T_A_* yields significant power savings compared with the conventional mobility management scheme for Mobile IP-based wireless networks.

### 3.3. Network Cost

We analyze the network cost due to registration and paging messages using the steady state probabilities. When an MH is in the *transmitting*, *receiving* or *attention/cell-connected* state, the MH performs a cell-based registration. If an MH is in the *attention/PA-connected* or *idle* state, the MH performs a PA-based registration, and the system can page the MH if data packets are destined for the MH. If an MH is not switched off, it performs a BL-based registration.

Let *ρ_MH_* and *A_tot_* denote the density of MHs within a total area and the size of the total area, respectively. The rates of cell-based registration, PA-based registration, BL-based registration, and paging messages for the proposed scheme are expressed as:

(26) $$\lambda_{\mathrm{prop}}^{\mathrm{cell}}=\rho_{\mathrm{MH}}A_{\mathrm{tot}}\left(P_{1}+P_{2}+P_{3}\right)\lambda_{c}$$

(27) $$\lambda_{\mathrm{prop}}^{\mathrm{PA}}=\rho_{\mathrm{MH}}A_{\mathrm{tot}}\left(P_{4}+P_{5}\right)\lambda_{\mathrm{PA}}=\rho_{\mathrm{MH}}A_{\mathrm{tot}}\left(P_{4}+P_{5}\right)\lambda_{c}/\sqrt{N_{\mathrm{cell}/PA}}$$

(28) $$\lambda_{\mathrm{prop}}^{\mathrm{BL}}=\rho_{\mathrm{MH}}A_{\mathrm{tot}}\sum_{i=1}^{5}P_{i}\frac{{\bar{N}}_{\mathrm{BL},i}}{{\bar{T}}_{i}}$$

(29) $$\lambda_{\mathrm{prop}}^{\mathrm{pag}}=\rho_{\mathrm{MH}}A_{\mathrm{tot}}\left(P_{4}+P_{5}+kP_{6}\right)\lambda_{i}N_{\mathrm{cell}/PA}$$

where *N_cell/PA_* is the number of cells in PA, *N̄_BL,i_* is the mean number of BL-based registrations during the mean sojourn time in state *i*, and *k* is the number of paging repetitions when the paging is unsuccessful. In Equation (27), λ*_PA_* is expressed with λ*_c_* and *N_cell/PA_* under the assumptions of a square shaped configuration of cell and PA, and a fluid flow mobility model of MHs [5], [22]. In Equation (29), $\lambda_{\mathrm{prop}}^{\mathrm{pag}}$ is expressed with *N_cell/PA_* because an additional paging cost is incurred as different cells in PA transmit the same paging messages.

We consider that MHs move at an average speed of *V_i_* according to the environment type which consists of stationary user environment (*i* = 0), urban in-building environment (*i* = 1), urban pedestrian environment (*i* = 2), and urban vehicular environment (*i* = 3) [23–25]. It is considered that MHs move in directions which are uniformly distributed over [0,2*π*], and the MHs are uniformly distributed with a density of *ρ_MH_*. The average number of MHs crossing out of the cell and the PA per unit time, *r_cell_* and *r_PA_*, respectively, are given by:

$$r_{\mathrm{cell}}=\frac{\rho_{\mathrm{MH}}l_{\mathrm{cell}}}{\pi }\sum_{i=0}^{3}P_{{\mathrm{env}}_{i}\;}V_{i},\;\;\;\;\;\;\;r_{\mathrm{PA}}=\frac{\rho_{\mathrm{MH}}l_{\mathrm{PA}}}{\pi }\sum_{i=0}^{3}P_{{\mathrm{env}}_{i}}V_{i},$$

where *l_cell_* and *l_PA_* are the length of the cell perimeter and the PA perimeter, respectively. The average rates of cell updates and PA updates of an MH are obtained by:

$$\begin{array}{c} \lambda_{c}=\frac{r_{\mathrm{cell}}}{{\left(\frac{l_{\mathrm{cell}}}{4}\right)}^{2}\rho_{\mathrm{MH}}}=\frac{16}{\pi l_{\mathrm{cell}}}\sum_{i=0}^{3}P_{{\mathrm{env}}_{i}}V_{i}\\ \lambda_{\mathrm{PA}}=\frac{r_{\mathrm{PA}}}{{\left(\frac{l_{\mathrm{PA}}}{4}\right)}^{2}\rho_{\mathrm{MH}}}=\frac{16}{\pi l_{\mathrm{cell}}\sqrt{N_{\mathrm{cell}/\mathrm{PA}}}}\sum_{i=0}^{3}P_{{\mathrm{env}}_{i}}V_{i} \end{array}$$

Let *r*_1_ be the time interval from the instant that the MH enters state *i* to the instant that the MH conducts the first BL-based registration in the state. Let *f*_*r*_1__ (*t*) and *f*_*T*_*i*__ (*t*) denote the density functions of the time interval *r*_1_ and the sojourn time *T_i_*, respectively. Then, the mean number of BL-based registrations *N̄_BL,i_* is obtained by:

(30) $$\begin{array}{l} {\bar{N}}_{\mathrm{BL},i}=\sum_{n=1}^{\infty }n\mathrm{Pr}\left[N_{\mathrm{BL},i}=n\right]\\ \;\;\;\;\;\;\;\;\;\;\;=\sum_{n=1}^{\infty }\frac{n}{2\pi j}\int_{\sigma -j\infty }^{\sigma +j\infty }\frac{F_{r_{1}}^{*}(\theta ){\left[F_{r}^{*}(\theta )\right]}^{n-1}\left[1-F_{r}^{*}(\theta )\right]}{\theta }F_{T_{i}}^{*}(-\theta )d\theta \end{array}$$

where $F_{r_{1}}^{*}(\theta )$ and $F_{T_{i}}^{*}(\theta )$ denote the Laplace transforms of *f*_*r*_1__ (*t*) and *f*_*T*_*i*__ (*t*), respectively. Equation (30) is evaluated using the Residue theorem [26]. We define a cost function as the weighted sum of the rates of cell-based registration, PA-based registration, BL-based registration, and paging messages for the proposed scheme as follows:

(31) $$C_{\mathrm{prop}}^{\mathrm{tot}}=C_{\mathrm{prop}}^{\mathrm{reg}}+C_{\mathrm{prop}}^{\mathrm{pag}}$$

(32) $$C_{\mathrm{prop}}^{\mathrm{reg}}=C_{\mathrm{prop}}^{\mathrm{cell}}+C_{\mathrm{prop}}^{\mathrm{PA}}+C_{\mathrm{prop}}^{\mathrm{BL}}=w_{\mathrm{cell}}\lambda_{\mathrm{prop}}^{\mathrm{cell}}+w_{\mathrm{PA}}\lambda_{\mathrm{prop}}^{\mathrm{PA}}+w_{\mathrm{BL}}\lambda_{\mathrm{prop}}^{\mathrm{BL}}$$

(33) $$C_{\mathrm{prop}}^{\mathrm{pag}}=w_{\mathrm{pag}}\lambda_{\mathrm{prop}}^{\mathrm{pag}}$$

where *w_cell_*, *w_PA_*, *w_BL_*, and *w_pag_* are weighting factors. If the registration interval and session holding time follow exponential distributions, an optimal value of the mean rate of BL-based registration $\lambda_{r}^{*}$ that minimizes $C_{\mathrm{prop}}^{\mathrm{tot}}$ is derived as:

(34) $$\lambda_{r}^{*}=-\left(\lambda_{i}+\mu_{\mathrm{off}}\right)+\sqrt{\frac{w_{\mathrm{pag}}k\lambda_{i}\lambda_{\mathrm{off}}N_{\mathrm{cell}/PA}}{w_{\mathrm{BL}}}}$$

The optimal rate of BL-based registration is determined by incoming session rate, the duration that an MH remains switched-off, the number of paging repetitions, the number of cells in PA, the switch-off rate, and the weighting factors *w_pag_* and *w_BL_*. We can also derive an optimal value of the number of cells in PA $N_{\mathrm{cell}/PA}^{*}$ that minimizes $C_{\mathrm{prop}}^{\mathrm{tot}}$ as:

(35) $$N_{\mathrm{cell}/\mathrm{PA}}^{*}={\left(\frac{w_{\mathrm{PA}}}{w_{\mathrm{pag}}}\frac{\lambda_{c}}{2\lambda_{i}}\frac{P_{4}+P_{5}}{P_{4}+P_{5}+kP_{6}}\right)}^{2/3}$$

where the sum of steady state probabilities *P*_4_ + *P*_5_ is derived from Equations (19) and (20).

(36) $$P_{4}+P_{5}=\frac{\lambda_{\mathrm{off}}\mu_{\mathrm{off}}}{\lambda_{\mathrm{off}}+\mu_{\mathrm{off}}}\frac{\left(\lambda_{1}+\lambda_{c}\right)e^{-\lambda_{1}}}{\lambda_{1}\left(\lambda_{1}+\lambda_{c}e^{-\lambda_{1}}\right)}{\left[1-\frac{\lambda_{s}}{\lambda_{s}+\lambda_{\mathrm{off}}}F_{s}^{*}\left(\lambda_{\mathrm{off}}\right)\right]}^{-1}$$

The optimal number of cells in PA is determined by incoming session rate, the cell-based registration rate, the number of paging repetitions, the weighting factors *w_PA_* and *w_pag_*, and the steady state probabilities *P*_4_ + *P*_5_ and *P*_6_.

Since the conventional MIP protocol [1–2] does not distinguish between active and idle MHs, the value of attention timer *T_A_* approaches *∞*. The rates of cell-based registration, BL-based registration, and paging messages for the conventional scheme are obtained as:

(37) $$\lambda_{\mathrm{conv}}^{\mathrm{cell}}=\rho_{\mathrm{MH}}A_{\mathrm{tot}}\left(P_{1}+P_{2}\underset{T_{A}\to \infty }{\text{lim}}P_{3}\right)\lambda_{c}=\rho_{\mathrm{MH}}A_{\mathrm{tot}}\sum_{i=1}^{5}P_{i}\lambda_{c}$$

(38) $$\lambda_{\mathrm{conv}}^{\mathrm{BL}}=\rho_{\mathrm{MH}}A_{\mathrm{tot}}\sum_{i=1}^{5}P_{i}\frac{{\bar{N}}_{\mathrm{BL},i}}{{\bar{T}}_{i}}$$

(39) $$\lambda_{\mathrm{conv}}^{\mathrm{pag}}=\rho_{\mathrm{MH}}A_{\mathrm{tot}}kP_{6}\lambda_{i}$$

If an MH is in the off/attached state, the MH does not respond to paging for incoming sessions. We consider this unsuccessful paging in the conventional MIP protocol. We define the network cost for the conventional scheme as the weighted sum of the rates of cell-based registration, BL-based registration, and paging messages as follows:

(40) $$C_{\mathrm{conv}}^{\mathrm{tot}}=C_{\mathrm{conv}}^{\mathrm{reg}}+C_{\mathrm{conv}}^{\mathrm{pag}}$$

(41) $$C_{\mathrm{conv}}^{\mathrm{reg}}=C_{\mathrm{conv}}^{\mathrm{cell}}+C_{\mathrm{conv}}^{\mathrm{BL}}=w_{\mathrm{cell}}\lambda_{\mathrm{conv}}^{\mathrm{cell}}+w_{\mathrm{BL}}\lambda_{\mathrm{conv}}^{\mathrm{BL}}$$

(42) $$C_{\mathrm{conv}}^{\mathrm{pag}}=w_{\mathrm{pag}}\lambda_{\mathrm{conv}}^{\mathrm{pag}}$$

From Equations (26–33) and (37–42), the relationship of the registration cost between the proposed and conventional schemes is expressed as $C_{\mathrm{prop}}^{\mathrm{reg}}\leq C_{\mathrm{conv}}^{\mathrm{reg}}$ if *w_cell_* = *w_PA_*. Since the conventional scheme considers only unsuccessful paging for the off/attached MHs, the relationship of the paging cost between the proposed and conventional schemes is obtained by $C_{\mathrm{prop}}^{\mathrm{pag}}\geq C_{\mathrm{conv}}^{\mathrm{pag}}$.

## 4. Numerical Examples

The effects of various input parameters on the steady state probabilities and power consumption of the proposed and conventional schemes are investigated. The values of input parameters assumed for numerical examples are shown in Table 1 [14–16], [27–29].

**Figure 3.**
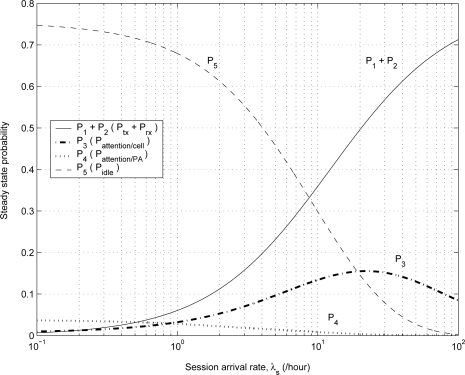
The effect of λ*_s_* on the steady state probabilities.

Figure 3 shows the effect of session arrival rate λ*_s_* on the steady state probabilities. The session arrival rate λ*_s_* is expressed as λ*_s_* = λ*_i_* + λ*_o_*. The higher the session arrival rate, the higher the transition probabilities *P*_31_, *P*_41_, *P*_51_ *P*_32_, *P*_42_, and *P*_52_ due to the high rate of incoming or outgoing session arrivals. Therefore, as the value of λ*_s_* increases, the probability *P*_1_+*P*_2_ increases, but the probability *P*_5_ decreases.

**Figure 4.**
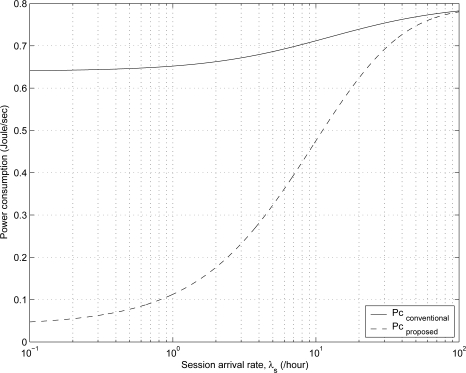
The comparison of the power consumption in the proposed and conventional schemes for varying the values of λ*_s_*.

Figure 4 shows the effect of session arrival rate λ*_s_* on the power consumption for the proposed and conventional schemes. The power consumption is calculated as the energy consumption divided by time. The energy consumption for the proposed and conventional schemes is calculated using Equations (23) and (25), respectively. The power consumption for the proposed and conventional schemes increases as the value of λ*_s_* increases because it is more likely that MHs stay in the *transmitting* and *receiving* states as the value of λ*_s_* increases. The MH power consumption in the proposed scheme is approximately 0.1126 W, 0.1763 W and 0.4756 W at the session arrival rate λ*_s_* = 1(/h), λ*_s_* = 2(/h), and λ*_s_* = 10(/h), respectively. The MH power consumption for the conventional scheme is approximately 0.6521 W, 0.6625 W and 0.7119 W at λ*_s_* = 1(/h), λ*_s_* = 2(/h), and λ*_s_* = 10(/h), respectively. Thus, for 2 *≤* λ*_s_* *≤* 10, the power consumption of the proposed scheme is reduced by about 33.2% ∼ 73.4% compared with the conventional scheme. Furthermore, if the session arrival rate is low (λ*_s_* *≤* 1), the proposed scheme can save about 82.7% of the battery power consumption at the MH compared with the conventional scheme in Mobile IP-based wireless networks.

**Figure 5.**
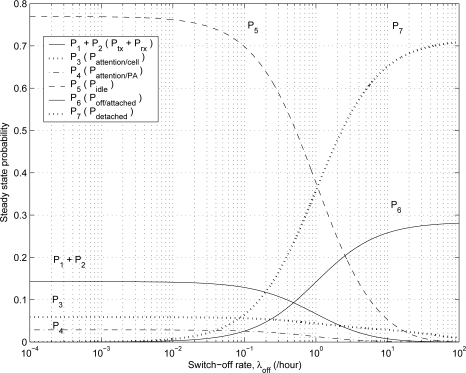
The effect of λ*_off_* on the steady state probabilities.

**Figure 6.**
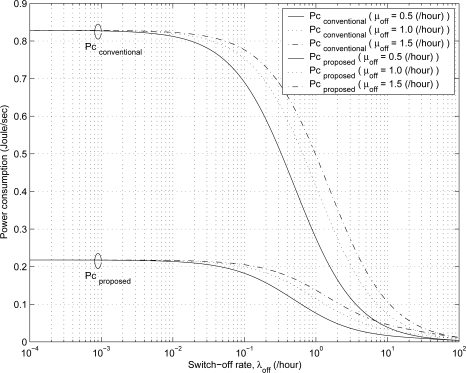
The comparison of the power consumption in the proposed and conventional schemes for varying the values of λ*_off_* and μ*_off_* .

Figure 5 shows the effect of switch-off rate λ*_off_* on the steady state probabilities. As the switch-off rate λ*_off_* increases, the probabilities *P*_6_ and *P*_7_ increase, but the probabilities *P*_1_, *P*_2_, *P*_3_, *P*_4_, and *P*_5_ decrease. Because it is more likely that MHs stay in the *off/attached* state and *detached* state as the switch-off rate λ*_off_* increases. Figure 6 compares the power consumption of the proposed scheme with that of the conventional scheme for varying the values of λ*_off_* and μ*_off_*. When an MH is in the *off/attached* state or *detached* state, the MH does not consume power because the MH is powered off. As the switch-off rate λ*_off_* increases and the mean switch-off duration 1/μ*_off_* increases, the power consumption for the proposed and conventional schemes decreases.

**Figure 7.**
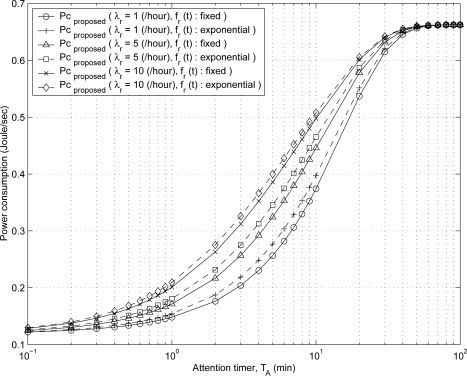
The effect of *T_A_* on the power consumption of the proposed scheme for various values of λ*_r_* if the interval of binding-lifetime-based registration is fixed (solid line) or exponentially (dashed line) distributed.

Figure 7 shows the effect of attention timer value *T_A_* on the power consumption of the proposed scheme for various values of BL-based registration rate λ*_r_* if the registration interval is fixed (solid line) or exponentially (dashed line) distributed. As the values of *T_A_* and λ*_r_* increase, power consumption of the proposed scheme increases because the probability *P*_5_ that an MH stays in the *idle* state decreases. An MH battery’s power is saved more as the value of attention timer *T_A_* decreases. However, the value of *T_A_* needs to be determined by considering the incoming session rate and the data session delay. The results show that the power consumption of the proposed scheme depends on the distribution of the registration intervals. The fixed registration intervals are better than the exponential registration intervals from the viewpoint of the power-saving effect of the proposed scheme.

**Figure 8.**
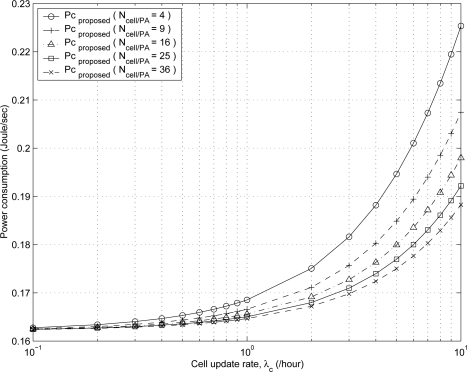
The effect of λ*_c_* on the power consumption of the proposed scheme for various values of *N_cell/PA_*.

Figure 8 shows the effect of cell update rate λ_*c*_ on the power consumption of the proposed scheme for various values of *N_cell/PA_*. As MH mobility increases, both the cell update rate λ_*c*_ and the power consumption increases because the probability *P*_5_ that the MH stays in the *idle* state decreases as the cell update rate λ_*c*_ increases. As the number of cells in PA *N_cell/PA_* decreases, both the PA update rate λ*_PA_* and the power consumption increases.

The effects of various input parameters on the network cost of the proposed scheme in IP-based wireless networks are investigated. The values of input parameters assumed for numerical examples are shown in Table 2 [23–25].

**Figure 9.**
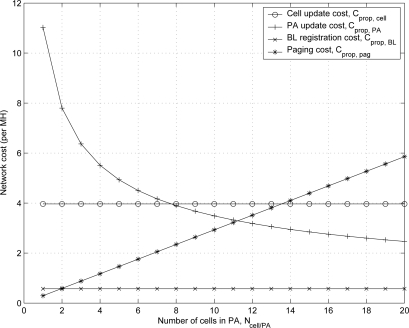
The effect of *N_cell/PA_* on the network cost of the proposed scheme.

Figure 9 shows the effect of *N_cell/PA_* on the network cost of the proposed scheme which consists of cell update cost $C_{\mathrm{prop}}^{\mathrm{cell}}$, PA update cost $C_{\mathrm{prop}}^{\mathrm{PA}}$, BL-based registration cost $C_{\mathrm{prop}}^{\mathrm{BL}}$, and paging cost $C_{\mathrm{prop}}^{\mathrm{pag}}$. As the number of cells in PA *N_cell/PA_* increases, the PA update cost decreases due to infrequent crossing out of the PA, while the paging cost increases due to paging messages for the large number of cells in PA. The cell update cost and the BL-based registration cost are fixed with various numbers of cells in PA *N_cell/PA_* according to Equations (26) and (28).

**Figure 10.**
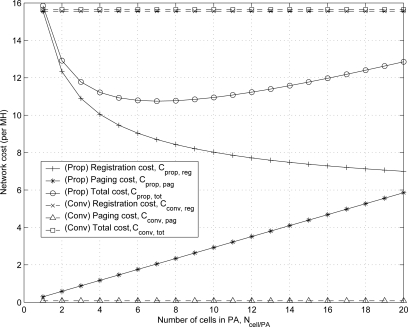
The optimal number of cells in paging area (PA) $N_{\mathrm{cell}/\mathrm{PA}}^{*}$ of the proposed scheme.

Figure 10 shows the effect of *N_cell/PA_* on registration cost $C_{\mathrm{prop}}^{\mathrm{reg}}$, $C_{\mathrm{conv}}^{\mathrm{reg}}$, paging cost $C_{\mathrm{prop}}^{\mathrm{pag}}$, $C_{\mathrm{conv}}^{\mathrm{pag}}$, and total network cost $C_{\mathrm{prop}}^{\mathrm{tot}}$, $C_{\mathrm{conv}}^{\mathrm{pag}}$ for the proposed and conventional schemes. The network costs for the conventional scheme are fixed with varying *N_cell/PA_* according to Equations (37–42). The numerical results show that $C_{\mathrm{prop}}^{\mathrm{pag}}\geq C_{\mathrm{conv}}^{\mathrm{pag}}$ and $C_{\mathrm{prop}}^{\mathrm{reg}}\leq C_{\mathrm{conv}}^{\mathrm{reg}}$ when *w_cell_* = *w_PA_*. For the proposed scheme, the optimal number of cells in PA $N_{\mathrm{cell}/PA}^{*}$ can exist. The result shows that total network cost with the input parameters is a minimum value at $N_{\mathrm{cell}/PA}^{*}=7$ which is consistent with Equation (35).

**Figure 11.**
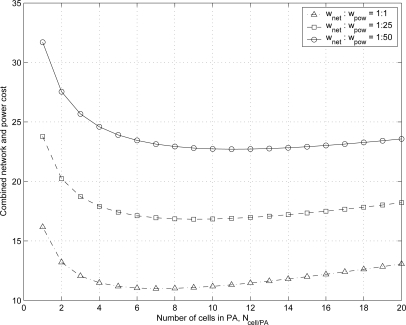
Combined network and power cost for the proposed scheme for varying the values of *N_cell/PA_*.

We define the combined network and power cost for the proposed scheme $C_{\mathrm{prop}}^{\mathrm{comb}}$ and that for the conventional scheme $C_{\mathrm{conv}}^{\mathrm{comb}}$ as:

(43) $$C_{\mathrm{prop}}^{\mathrm{comb}}=w_{\mathrm{net}}C_{\mathrm{prop}}^{\mathrm{tot}}+w_{\mathrm{pow}}Pc_{\mathrm{prop}}=w_{\mathrm{net}}C_{\mathrm{prop}}^{\mathrm{tot}}+w_{\mathrm{pow}}E_{\mathrm{prop}}/t$$

(44) $$C_{\mathrm{conv}}^{\mathrm{comb}}=w_{\mathrm{net}}C_{\mathrm{conv}}^{\mathrm{tot}}+w_{\mathrm{pow}}{\mathrm{Pc}}_{\mathrm{conv}}=w_{\mathrm{net}}C_{\mathrm{conv}}^{\mathrm{tot}}+w_{\mathrm{pow}}E_{\mathrm{conv}}/t$$

where *w_net_* and *w_pow_* are weighting factors for network cost and power consumption, respectively. Figure 11 shows the combined network and power cost $C_{\mathrm{prop}}^{\mathrm{comb}}$ for the proposed scheme with varying the values of $N_{\mathrm{cell}/\mathrm{PA}}^{*}$ and *w_pow_*. As the weighting factor *w_pow_* increases as 1,25 and 50 with fixed value of *w_net_*, the optimal number of cells in PA $N_{\mathrm{cell}/\mathrm{PA}}^{*}$ increases as 7, 9, and 11 for the combined network and power cost. It is because the MH power consumption for the proposed scheme decreases as *N_cell/PA_* increases as shown in Figure 8.

**Figure 12.**
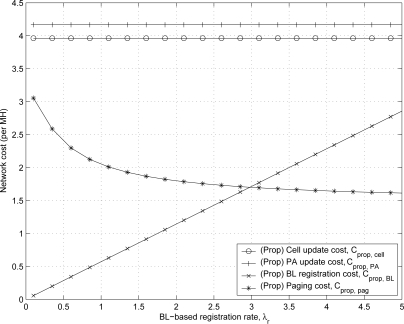
The effect of λ*_r_* on the network cost of the proposed scheme.

Figure 12 shows the effect of mean rate of BL-based registration λ*_r_* on the network cost of the proposed scheme which consists of cell update cost $C_{\mathrm{prop}}^{\mathrm{cell}}$, PA update cost $C_{\mathrm{prop}}^{\mathrm{PA}}$, BL-based registration cost $C_{\mathrm{prop}}^{\mathrm{BL}}$, and paging cost $C_{\mathrm{prop}}^{\mathrm{pag}}$. As the BL-based registration rate λ*_r_* increases, the BL-based registration cost increases and the paging cost decreases due to the frequent BL-based registrations. The rates of cell-based registration and PA-based registration are fixed with various values of λ*_r_* according to Equations (26) and (27).

**Figure 13.**
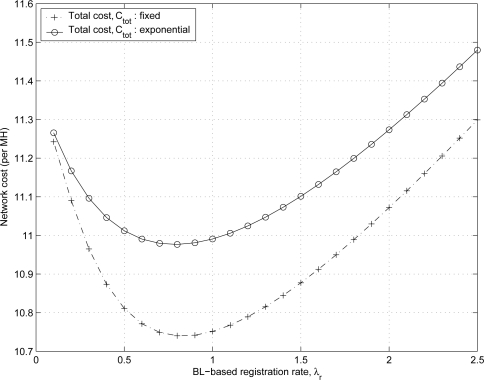
The optimal binding-lifetime-based registration rate $\lambda_{r}^{*}$ of the proposed scheme.

Figure 13 shows the effect of λ*_r_* on the total network cost *C_tot_* and the optimal BL-based registration rate $\lambda_{r}^{*}$ for two types of distributions of registration intervals, exponential (dashed line) and fixed (solid line) distributions. The result shows that the distribution of the BL-based registration intervals affects the network cost. If the BL-based registration intervals follow an exponential distribution, the total network cost *C_tot_* is a minimum value of 10.98 at $\lambda_{r}^{*}=0.8$ which is consistent with Equation (34). When the fixed BL-based registration intervals are utilized, the minimum network cost is 10.74 at $\lambda_{r}^{*}=0.8$. Therefore, the fixed BL-based registration intervals are preferred over the exponential BL-based registration intervals since the fixed BL-based registration intervals yield a lower network cost in the proposed scheme for IP-based wireless networks.

**Figure 14.**
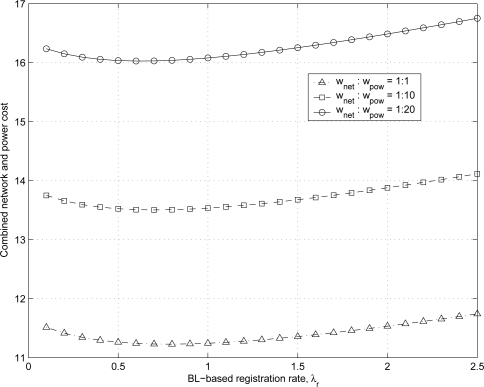
Combined network and power cost of the proposed scheme for varying the values of λ*_r_*.

Figure 14 shows the combined network and power cost $C_{\mathrm{prop}}^{\mathrm{comb}}$ for the proposed scheme with exponential BL-based registration intervals for various values of λ*_r_* and *w_pow_*. As shown in Figure 7, the power consumption of MHs for the proposed scheme increases as λ*_r_* increases. Hence, as the weighting factor *w_pow_* increases as 1, 10, and 20 with fixed value of *w_net_*, the optimal BL-based registration rate $\lambda_{r}^{*}$ for the combined network and power cost decreases as 0.8, 0.7, and 0.6.

## 5. Conclusions

An energy-efficient mobility management scheme was proposed to reduce MH power consumption in IP-based wireless networks. The proposed scheme manages the following seven MH states, including transmitting, receiving, attention/cell-connected, attention/PA-connected, idle, off/attached, and detached states, to efficiently manage battery power, radio resources, and network load. The MH state transition behavior was modeled. We derived the stationary probabilities and steady state probabilities of the MH states for the proposed and conventional schemes in IP-based wireless networks in compact form. The effects of various input parameters on the MH steady state probabilities and power consumption were investigated in the proposed scheme compared to the conventional scheme with consideration of exponential and fixed distributions on interval of BL-based registration. The proposed scheme yielded significant power savings compared with the conventional mobility management scheme for Mobile IP based wireless networks. Network costs such as cell updates, PA updates, BL-based registrations, and paging messages were analyzed in the proposed and conventional schemes for IP-based wireless networks. The optimal values of PA size and registration interval were derived to minimize the network cost of the proposed scheme. The effects of various input parameters on the network cost were investigated. We also investigated the combined network and power cost with various weighting factors for the proposed and conventional schemes. These analytical results provide guidelines to select proper system parameters. The results can be utilized to analyze the performance of mobility management schemes in IP-based wireless networks.

## Figures and Tables

**Table 1.** Input Parameters.

**Parameter**	**Value**
λ*_i_*	1 (/h)
λ*_o_*	1 (/h)
*μ_s_*	3,600/300 (/h)
λ*_c_*	4 (/h)
λ*_PA_*	$\lambda_{c}/\sqrt{N_{\mathrm{cell}/PA}}(/h)$
*N_cell/PA_*	16
*T_A_*	120/3,600 (h)
λ*_r_*	1 (/h)
λ*_off_*	1/4 (/h)
*μ_off_*	1 (/h)
*Pc*_1_	1.10 (W)
*Pc*_2_	0.90 (W)
*Pc*_3_	0.80 (W)
*Pc*_4_	0.70 (W)
*Pc*_5_	0.01 (W)
*Pc*_6_	0 (W)
*Pc*_7_	0 (W)

**Table 2.** Input Parameters.

**Parameter**	**Value**	**Parameter**	**Value**
λ*_i_*	1/2 (/h)	*λ_o_*	1/2 (/h)
*μ_s_*	10 (/h)	*T_A_*	1/15 (h)
λ*_off_*	1/4 (/h)	*μ_off_*	1/3 (/h)
*T_r_*	1 (h)	$w_{\mathrm{reg}}^{\mathrm{cell}}$, $w_{\mathrm{reg}}^{\mathrm{PA}}$	1
*k*	3	$w_{\mathrm{reg}}^{\mathrm{BL}}$, *w_pag_*	1
*ρ_MH_*	100 (/*km*^2^)	*l_cell_*	2 (*km*)
*V*_0_	0 (*km/hr*)	*P_env_*_0_	0.40
*V*_1_	2 (*km/hr*)	*P_env_*_1_	0.25
*V*_2_	4 (*km/hr*)	*P_env_*_2_	0.20
*V*_3_	60 (*km/hr*)	*P_env_*_3_	0.15
